# Robotic-Assisted Conversion of Unicompartmental Knee Arthroplasty to Total Knee Arthroplasty: A Surgical Technique Review

**DOI:** 10.1016/j.artd.2025.101748

**Published:** 2025-06-17

**Authors:** Harold I. Salmons, Andrew D. Pumford, Nicholas A. Bedard, Charles P. Hannon, Cameron K. Ledford

**Affiliations:** a Department of Orthopedic Surgery, Mayo Clinic, Rochester, MN, USA; b Department of Orthopedic Surgery, Mayo Clinic, Jacksonville, FL, USA

**Keywords:** Bone loss management, Surgical planning, Fixation strategies, Alignment

## Abstract

Conversion of a failed unicompartmental knee arthroplasty (UKA) to total knee arthroplasty (TKA) is challenged by asymmetric bone loss necessitating advanced fixation strategies and alignment considerations. Robotic-assisted TKA is an increasingly utilized tool to augment preoperative surgical planning, improve intraoperative execution, and optimize patient outcomes. However, the use of robotic-assisted TKA in conversion UKA is not well described in the literature. We present the authors' preferred surgical technique of conversion UKA utilizing robotic assistance with a haptically guided robotic system and present a case example of a patient managed with this technique.

## Introduction

Unicompartmental knee arthroplasty (UKA) is a surgical option for the management of osteoarthritis in a single compartment of the knee. While most UKAs are performed for medial compartment osteoarthritis, 10% of patients have isolated lateral compartment disease [1]. When compared to total knee arthroplasty (TKA), UKAs offer the benefits of being less invasive, leading to improved postoperative pain, and potentially superior early functional improvements [[2], [3], [4]]. However, historical implant survivorship free from revision is less than ideal, particularly in younger patients [5]. Existing data suggest that revision rates of UKAs are at least 2 times higher than TKAs, and many of these revisions involve conversion TKA due to progression of osteoarthritis in the initially uninvolved knee compartments [3,[5], [6], [7], [8], [9]].

The most common failure modes of UKA are instability, progression of osteoarthritis in other compartments, and aseptic tibial component loosening [[10], [11], [12], [13]]. When revision is indicated, UKAs are typically converted to TKA [9,14,15]. Conversion of failed UKAs to TKA are technically challenging procedures given bone loss, alignment and balance considerations, and the need to achieve a stable construct in the setting of previous surgery [16,17].

Robotic-assisted TKA represents an increasingly utilized tool to assist surgeons with enhanced preoperative planning, intraoperative control, intraoperative decision-making, and perhaps improved outcomes. In the setting of UKA conversion to TKA, the authors believe that robotic-assisted TKA may lessen the amount of bone removed, reduce the need for adjuvant fixation such as augments, and more accurately restore mechanical alignment due to more precise planning and surgical execution. We present our preferred technique of robotic-assisted conversion TKA for failed UKA using the MAKO total knee system (Stryker, Mahwah, NJ, USA). Patient consent was obtained for publication.

## Surgical technique

A 42-year-old otherwise healthy male presented with a painful left medial (mobile-bearing) UKA performed 1 year prior. Past left knee surgical history was significant for 2 osteochondral allograft transplantation procedures 6 years prior to his UKA. Postoperative courses were complicated by stiffness and lack of terminal extension requiring 2 separate open lysis of adhesions that were unsuccessful in relieving his symptoms. It was in the setting of these numerous operations and ongoing pain and debility that he presented for a second opinion.

**Figure 1:**
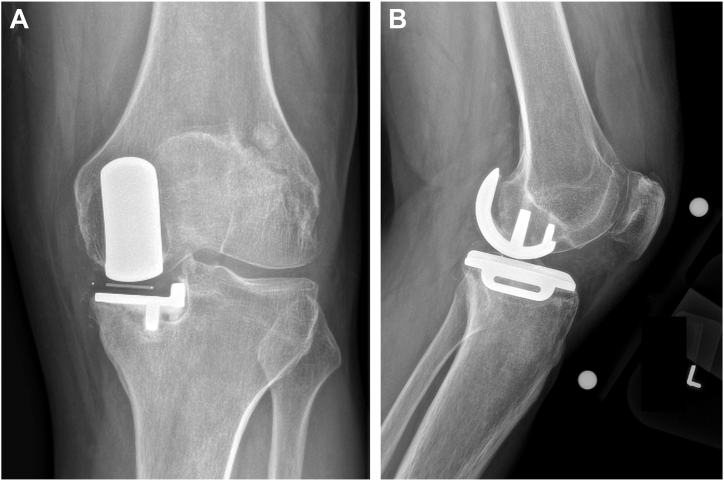
Preoperative anteroposterior (a) and lateral (b) radiograph of a failed unicompartmental knee arthroplasty.

At the time of presentation, the patient ambulated with an antalgic gait. His skin was intact and revealed a well-healed midline incision from his prior UKA. His knee range of motion was 5-120°. The knee was stable to anterior and posterior stress at full extension and 90 degrees of flexion and was also stable in varus/valgus stress at full extension, mid-flexion, and 90 degrees of flexion. However, he did endorse pain to valgus stress of the knee localized over the medial joint line. Radiographs demonstrated medial subluxation of the mobile bearing in flexion, tibial component malposition in valgus and femoral component malrotation (Fig. 1). The limb was overall in about 5° of varus. Serum erythrocyte sedimentation rate and C-reactive protein were obtained and within normal limits. As such, infection was ruled out, and the patient was subsequently indicated for conversion of their UKA to TKA to relieve pain, restore alignment, and return to function.

Preoperatively, a computed tomography scan was obtained using the MAKO robotic-assisted TKA protocol (Stryker, Mahwah, USA). Preoperative alignment considerations targeted a functional mechanical alignment using a posterior stabilized implant with a tibial resection perpendicular to the mechanical axis of the tibia matching native slope, and a femoral distal valgus resection of 5 degrees from the anatomical axis.

**Figure 2:**
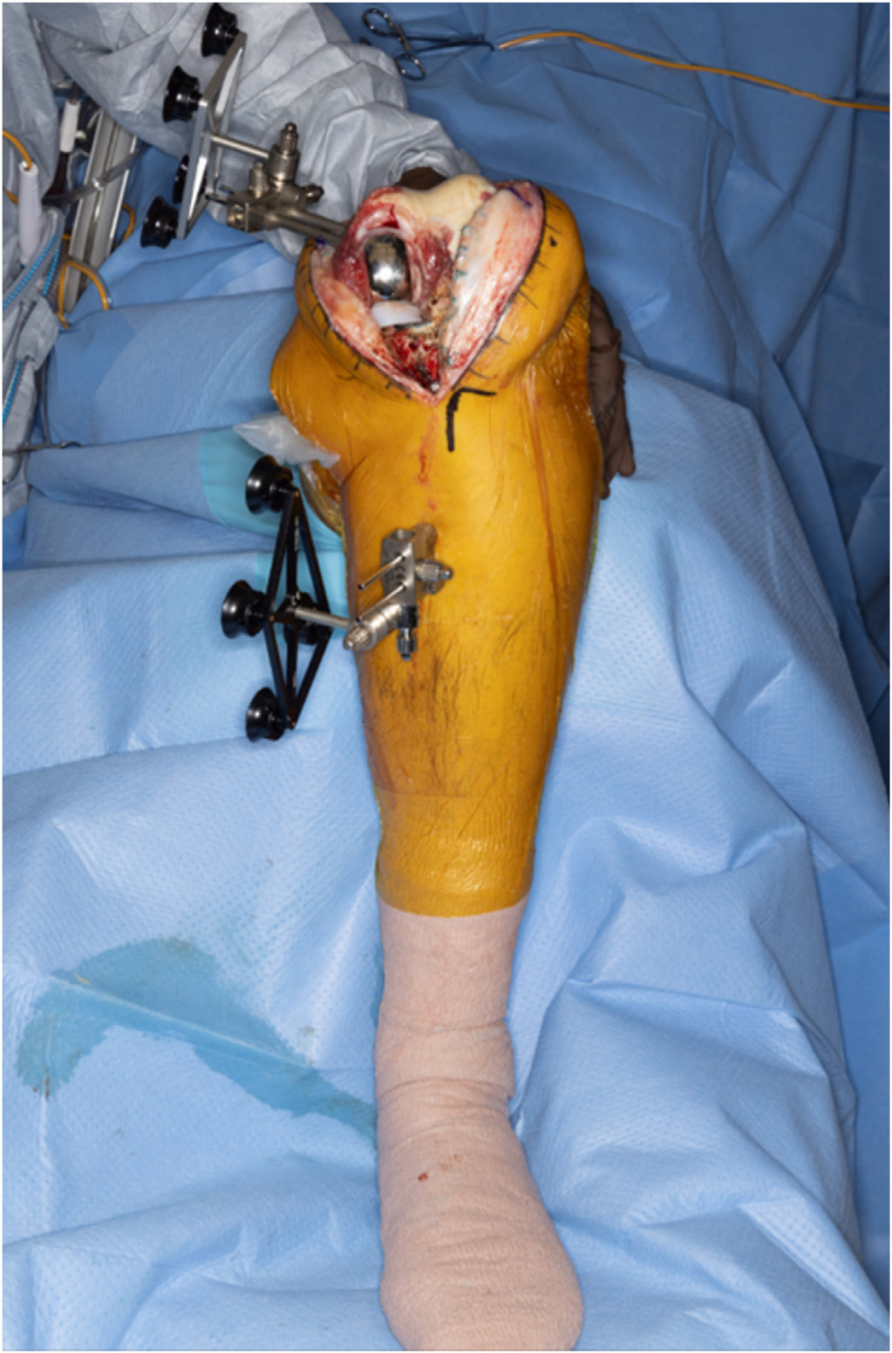
The author's preferred technique of reference frame position.

**Figure 3:**
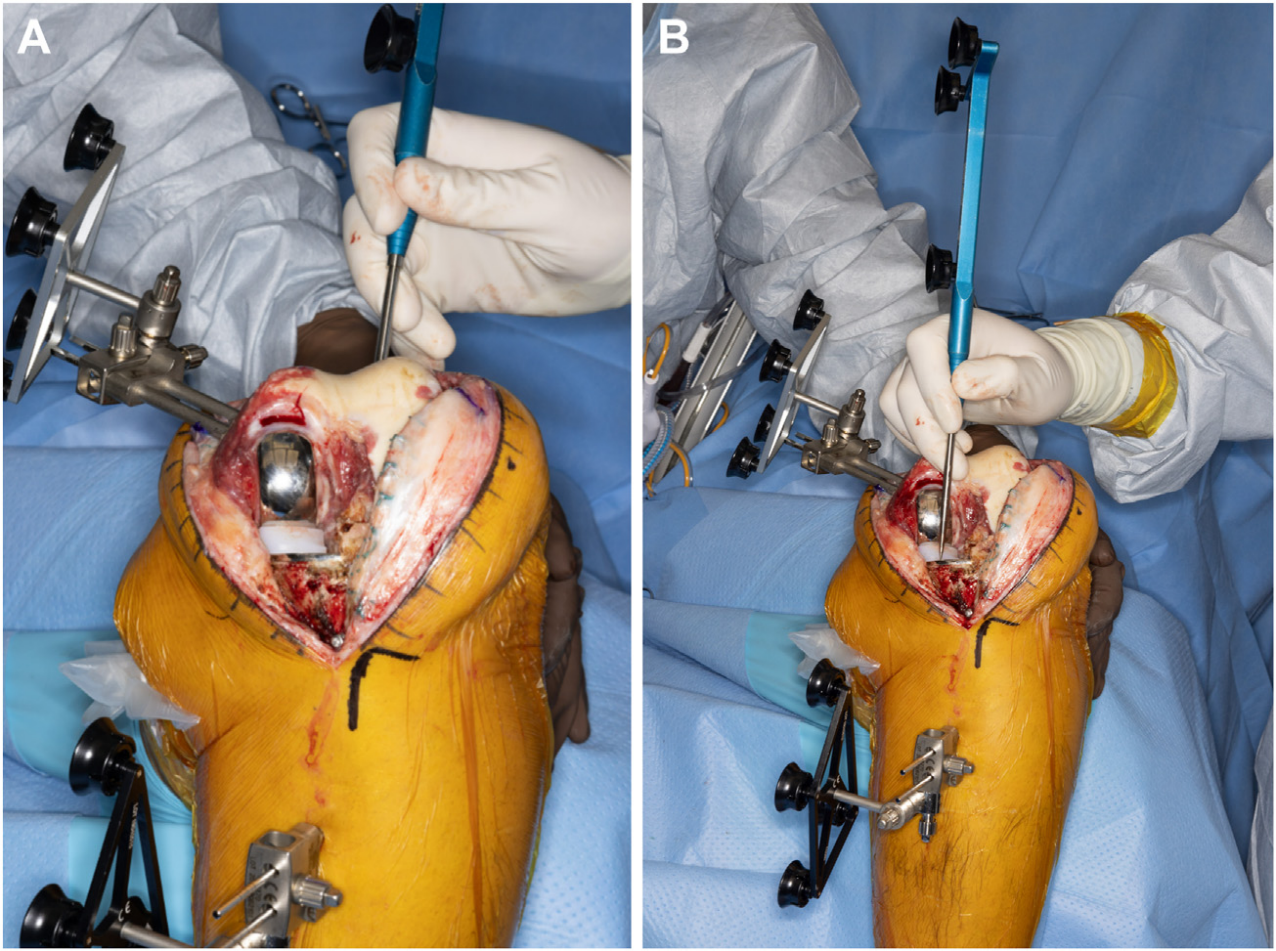
Bone registration. A sharp probe is used following on-screen instructions to register 40 individual osseous points along the distal femur (a) and proximal tibia (b). Note that the probe was directed along the metal tray and not the polyethylene liner of the tibial component. This permits robotic registration of the 3-dimensional anatomy previously ascertained by computed tomography scan preoperatively.

**Figure 4:**
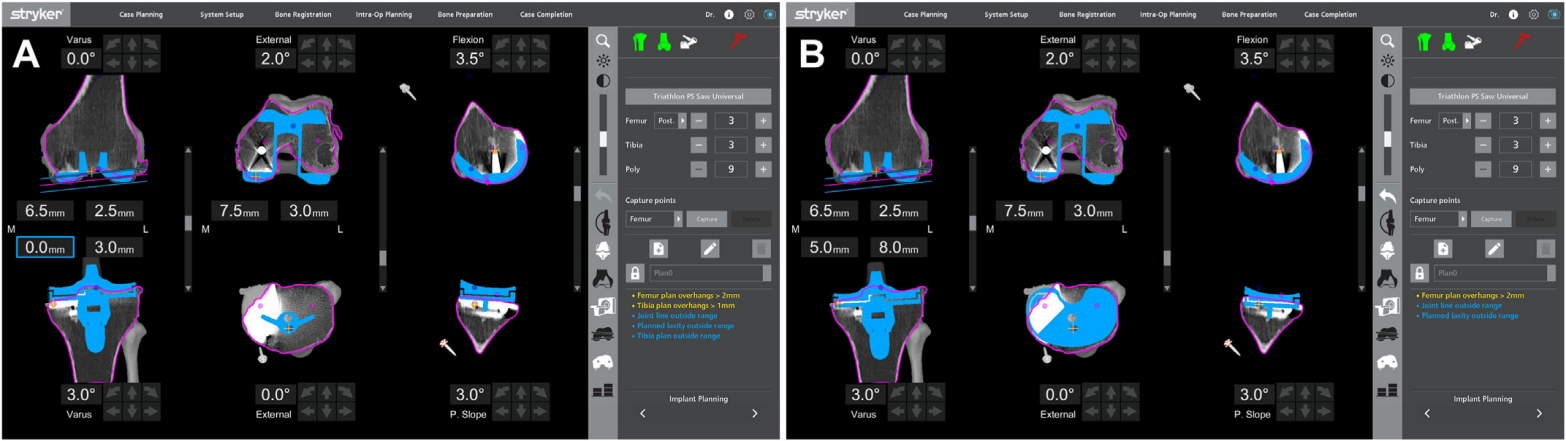
Preoperative MAKO registration plan. (a) Demonstrates an anticipated 0-mm resection off of the medial proximal tibia with a 3-degree varus angle selected to optimize functional balance of the knee. (b) Demonstrates how the MAKO system can be implemented to accommodate a 5 mm augment by dropping the medial tibia resection depth by 5 mm. This technique was not necessary for the present case.

A standard midline parapatellar approach was undertaken. Inspection of the knee revealed lateral and patellofemoral compartment arthritis. A standard release of the superficial medial collateral ligament from the proximal medial tibia was performed to the mid-sagittal line. Scar was cleared from the medial and lateral gutters to facilitate flexion and forward translation of the tibial component. Threaded pins were then inserted percutaneously into the proximal medial tibia and intraincisional within the medial distal femur. The authors' preferred position of the distal medial femoral pins is approximately 1 centimeter anterior and proximal to the medial epicondyle, placed at a 45-degree angle to the long axis of the femur and parallel to the joint line. Reference frames were then attached to the pins and oriented toward the robot censor (Fig. 2). In standard fashion, the limb was then cycled through a full range of motion to register the hip center of rotation, checkpoints on both the femur and tibia arrays were registered, and then the on-screen system was referenced while using a sharp probe to identify 40 individual osseous points along the distal femur and proximal tibia to register the bone in 3-dimensions (Fig. 3). While the sequence of knee registration was performed pursuant to standard MAKO protocols, the knee registration was performed with the original medial UKA left in place. The computer system was then referenced to verify accuracy of registration to within 2 mm. After verifying accuracy of the registration, osteophytes, menisci, and the anterior and posterior cruciate ligaments were resected, and gaps were assessed using the computer software. The final plan was registered, and the computer arm was brought into the operative field to begin bone resections (Fig. 4). Note, while not necessary in the present case, if the surgeon feels that an augment is necessary, the MAKO plan can be adjusted by increasing resection depth by 5 mm to accommodate said augment. Tibial slope was set to 3 degrees in final registration to accommodate the anticipated posterior-stabilized implant.

**Figure 5:**
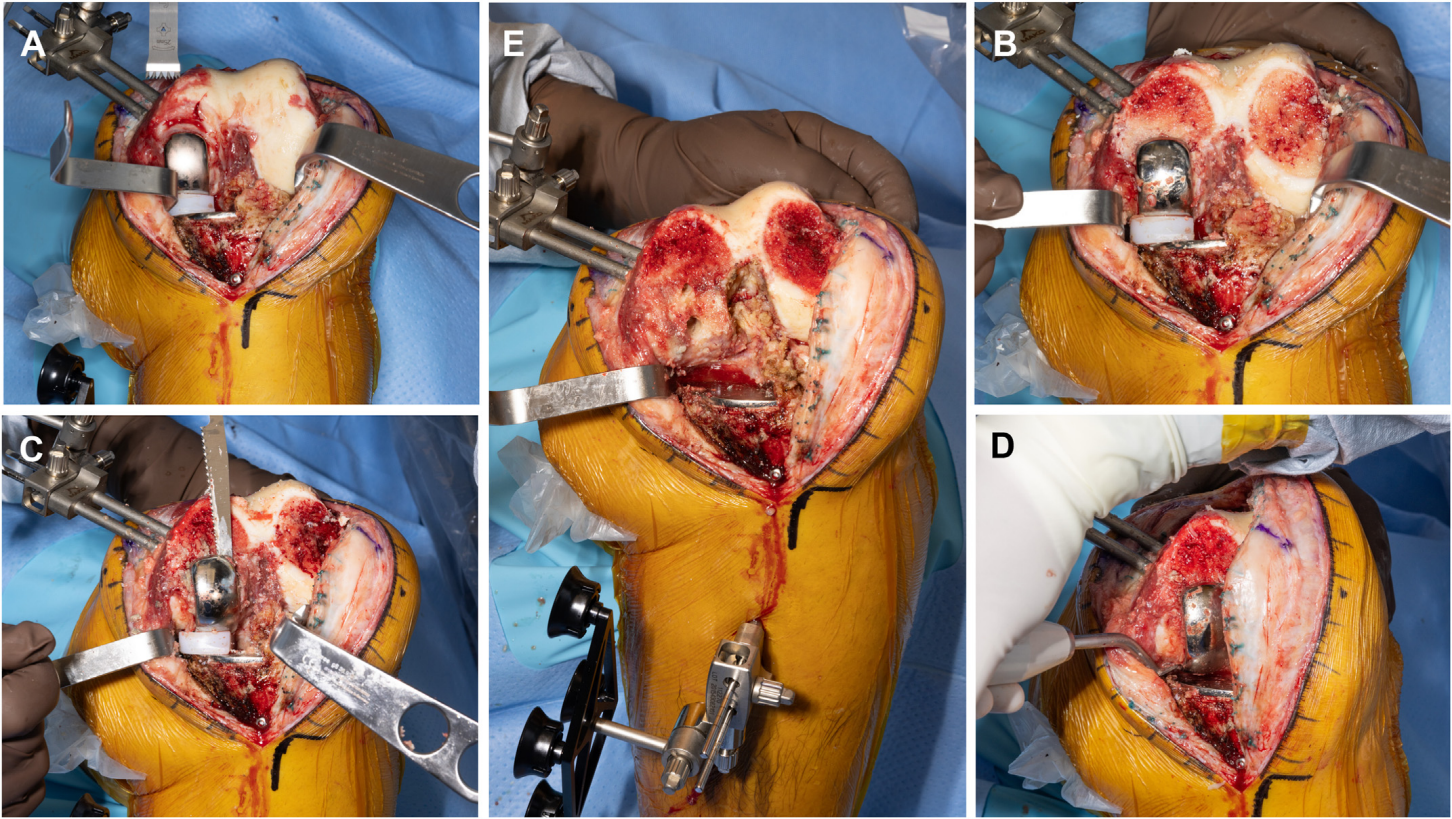
Sequence of distal femur osteotomy and femoral component removal. (a) The distal femur resection is started with the saw advanced through the femoral component medially and through the lateral distal femur laterally. The saw at this stage is used to disrupt the cement-bone interface of the femoral component medially and completing the distal lateral femoral osteotomy laterally (b). (c) A single-sided reciprocating saw is also helpful to further work the cement-bone interface of the femoral component medially and laterally. (d) A curved osteotome is used to score the posterior condylar cement-bone interface in order to prevent bone loss in this area when disimpacting the component. Following the above steps can help minimize bone loss of the distal femur (e).

**Figure 6:**
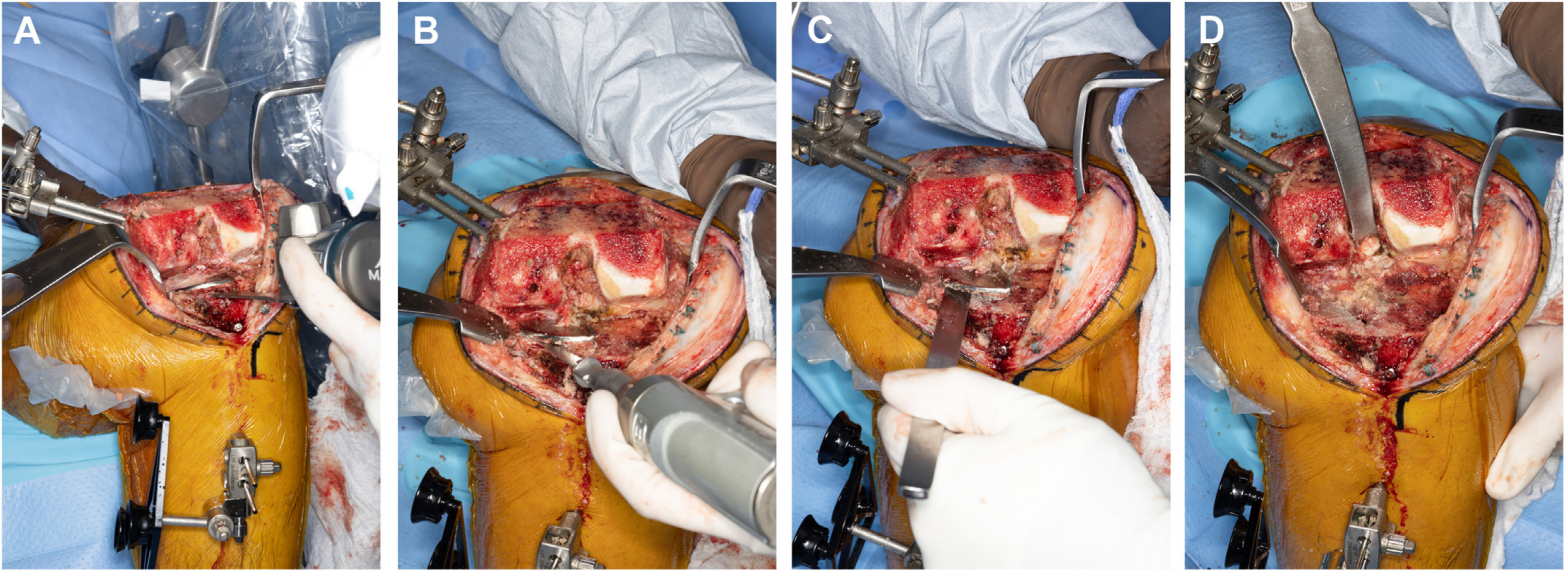
Tibial osteotomy and component removal. (a) The proximal tibia is exposed with retractors and the robotic arm and reciprocating saw are introduced medially, beneath the tibial component, at the cement-bone interface. (b) A sagittal saw is used to complete the resection medially and is also used to disrupt the notch. (c) A ¾ inch osteotome is then implemented followed by an MK punch to disimpact the tibial component. Careful execution of this technique can lead to minimal medial proximal tibia bone loss (d). The remaining lateral tibial plateau is then resected in accordance with the preoperative plan.

The first resection was the distal medial femoral resection, with the saw advanced to the femoral component medially and through the lateral distal femur laterally (Fig. 5). The authors favor initiating the distal medial femoral cut with the medial UKA femoral component left in place in order to help set alignment while concomitantly facilitating implant removal. The saw was used to disrupt the cement-bone interface of the femoral component. A ¾ inch osteotome and a square tip impactor were then used to remove the femoral component. There was minimal bone loss. The remaining femoral resections were then made using the MAKO in standard fashion. Attention was then turned to the tibia, resection was started medially, using the MAKO saw to disrupt the bone-cement interface of the tibial component (Fig. 6). A sagittal saw was then used to disrupt the notch. A spiked impactor as described above was then used along with osteotomes to remove the tibial component. At this point, the remaining tibial lateral plateau was resected in accordance with the preoperative plan, and then trial components were placed verifying that a balanced knee was achieved. After satisfactorily achieving a balanced knee, the final posterior-stabilized implants were then cemented in place in standard fashion and the wound closed in layers.

Written informed consent was obtained for publication of the present deidentified surgical technique.

### Follow-up

**Figure 7:**
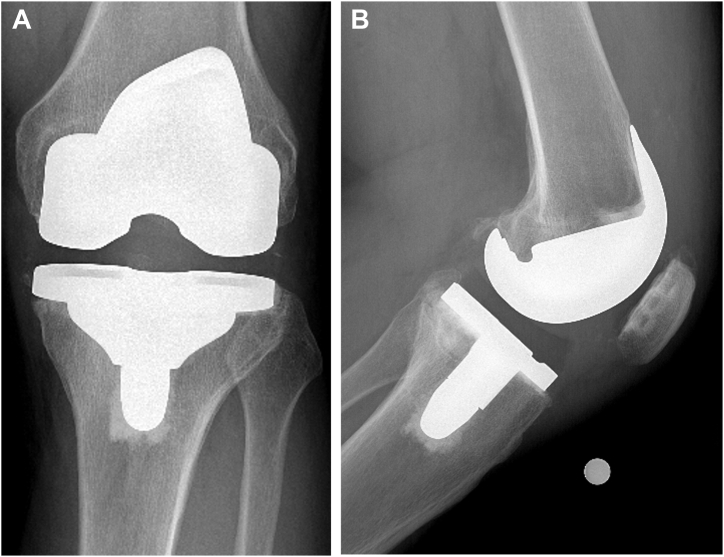
Final anteroposterior (a) and lateral (b) radiographs of a left conversion total knee arthroplasty for a failed unicompartmental knee arthroplasty at 1 year postoperative.

At their 1-year postoperative visit, the patient denied any complaints with their conversion TKA. Passive range of motion was from 0 to 120 degrees. The knee was stable in all planes, and radiographs demonstrated excellent fixation and alignment (Fig. 7).

## Discussion

Conversion TKA is the most common revision construct for failed UKAs, occurring at a rate of up to 40%, according to one large US database study [9,14,15,18]. When compared to primary TKAs, conversion TKAs are technically more challenging, with bone loss, alignment, and fixation options as primary considerations [16,17]. The present case report detailed the authors' preferred technique in performing conversion TKAs with the use of a haptically guided computed tomography–based robotic platform. We demonstrate an efficacious use of this system as an adjunct to UKA conversions with satisfactory results at the final follow-up.

In 2024, 3.7% of all arthroplasties submitted to the American Joint Replacement Registry were UKAs [19]. This proportion has remained stable over the years, with the total number of surgeons performing UKA having steadily increased from 200 in 2012 to 1300 in 2023 (25%) [19]. Revision risk remains a major consideration, with aseptic component loosening (36%) and progression of osteoarthritis (20%) contributing to the majority of these revisions [10]. While many caution against the widespread implementation of UKA in their practices for these reasons, others argue that newer implants, robotic assistance, and higher surgeon UKA volume through broadened indications may mitigate those risks and instead provide more optimal outcomes than TKA in select patients [3,20]. Additionally, the indications for UKA have continued to evolve and expand, reflecting an ongoing interest toward optimizing surgical techniques that appropriately address unicompartmental disease of the knee while maximizing function and minimizing complications and morbidity. Given these expanding concepts, UKA use in North America is not disappearing and will continue to contribute to revision scenarios that can be made simple if approached in a systematic way.

The outcomes of UKA conversions have been described. Leta et al. [21] compared revision TKAs from TKAs to conversion TKAs from failed UKAs using the Norwegian Arthroplasty Registry. In over 1000 knees assessed, the authors determined that the overall rerevision risk and surgical outcomes were similar between these groups. El-Galaly et al. [22] took this one step further and compared conversion of a failed UKA to TKA to both primary and revision TKAs. In their report of a Danish arthroplasty registry cohort, TKAs converted from medial UKA had a 3-fold higher risk of revision when compared with primary TKA. Notably, predominant aseptic failure modes included loosening (38% in conversion TKAs vs 27% in both de novo TKAs and revision TKAs) as well as instability (26% in conversion TKAs vs 16% and 18% in de novo and revision TKAs, respectively). As such, performing conversion TKAs needs to be approached in a thoughtful and well-planned manner in order to achieve optimal soft tissue balance and optimize outcomes.

In the present investigation, we present a stepwise approach to the conversion of a failed UKA to a TKA with the assistance of a haptically guided robotic navigation system. In our experience, the advantages of robotic navigation in cases such as this is to provide enhanced preoperative planning which helps improve intraoperative efficiency (this case's duration was 100 minutes) and minimize bone loss, particularly with the distal femur and proximal tibia resections. This technique is similar to that which was previously described by Piuzzi and colleagues [23] with the exception that our preference is to include the metallic UKA implants (with the polyethylene removed) when performing the robotic bone registration. We also routinely favor the use of a posterior-stabilized construct to optimize tracking, unless a more constrained construct is needed [23,24]. Additionally, while we favor a femur-first, functional alignment/modified measured resection technique, others have described a tibia-first, gap-balanced technique [25]. These cases cumulatively highlight how robotic assistance may help optimize preoperative planning by helping determine postoperative component position, limb alignment, and soft tissue balance. While robotic-assisted conversion TKAs have not been shown to be superior to manual conversions, the use of robotics in TKA at large has been demonstrated to significantly improve surgeon ability to execute preoperative alignment targets, minimize soft tissue resections, and potentially positively impact early pain scores [[25], [26], [27], [28]].

## Summary

Like any revision arthroplasty procedure, a systematic approach to conversion TKA for failed UKA is paramount to a positive outcome. Navigation and robotic-assisted technology provide a means of enhancing preoperative planning, alignment control, and intraoperative case flow that we feel anecdotally leads to improved outcomes for our patients. While this case describes a successful implementation of a haptically guided robotic system for the purposes of conversion TKA for a failed UKA, further study should explore whether robotic-assisted surgery may lead to an objective improvement in patient outcomes, knee kinematics, and implant survivorship following conversion TKA for failed UKA.

## Conflicts of interest

N.A. Bedard is a paid consultant for Stryker and Depuy; received research support from AAHKS FARE Grant as a Principal Investigator; and is a board member/committee appointments for AAHKS Evidence Based Medicine Committee and MAOA Education Committee. C.P. Hannon received royalties from Zimmer Biomet; is a paid consultant for Orchard Medical, Signature Orthopaedics, Enovis, and Vertex Pharmaceuticals; and is a board member/committee appointments for AAHKS, MAOA, and AAOS. C.K. Ledford is a paid consultant for Stryker; and is a board member/committee appointments for AAHKS, AAOS, and ABOS; all other authors declare no potential conflicts of interest.

For full disclosure statements refer to https://doi.org/10.1016/j.artd.2025.101748.

## CRediT authorship contribution statement

**Harold I. Salmons:** Writing – review & editing, Writing – original draft, Investigation, Formal analysis, Data curation. **Andrew D. Pumford:** Writing – review & editing, Investigation. **Nicholas A. Bedard:** Writing – review & editing. **Charles P. Hannon:** Writing – review & editing, Supervision. **Cameron K. Ledford:** Writing – review & editing, Supervision, Conceptualization.

## Appendix A Supplementary data

Conflict of Interest Statement for Ledford

Conflict of Interest Statement for Hannon

Conflict of Interest Statement for Pumford

Conflict of Interest Statement for Salmons

Conflict of Interest Statement for Bedard
