# Supplementary material for: Robotic-Assisted Conversion of Unicompartmental Knee Arthroplasty to Total Knee Arthroplasty: A Surgical Technique Review
Source: Arthroplast Today. 2025 Jun 17;34:101748. doi: 10.1016/j.artd.2025.101748 (PMC12209973; doi:10.1016/j.artd.2025.101748)
Supplement: Conflict of Interest Statement for Hannon [file mmc2.pdf]

CONFLICT OF INTEREST STATEMENT

American Association of Hip and Knee Surgeons

(Adopted from the American Academy of Orthopaedic Surgeons disclosure statement)

The following form **must be filled out completely and submitted by each author (example, 6 authors, 6 forms). All items require a response. If there is no relevant disclosure for a given item, enter "*None*."**

Manuscript Title: Robotic-Assisted Conversion of Unicompartmental Knee Arthroplasty to Total Knee Arthroplasty – Surgical Technique

1. Royalties from a company or supplier (The following conflicts were disclosed)

   Zimmer Biomet

2. Speakers bureau/paid presentations for a company or supplier (The following conflicts were disclosed)

   None

3A. Paid employee for a company or supplier (The following conflicts were disclosed)

   None

3B. Paid consultant for a company or supplier (The following conflicts were disclosed)

   Orchard Medical, Signature Orthopaedics, Enovis, Vertex Pharmaceuticals

3C. Unpaid consultants for a company or supplier (The following conflicts were disclosed)

   None

4. Stock or stock options in a company or supplier (The following conflicts were disclosed)

   None

5. Research support from a company or supplier as a Principal Investigator (The following conflicts were disclosed)

   None

6. Other financial or material support from a company or supplier (The following conflicts were disclosed)

   None

7. Royalties, financial or material support from publishers (The following conflicts were disclosed)

   None

8. Medical/Orthopaedic publications editorial/governing board (The following conflicts were disclosed)

   None

9. Board member/committee appointments for a society (The following conflicts were disclosed)

   AAHKS, MAOA, AAOS

Each author must sign AND print or type his/her name, date and submit a separate form

In addition, one BLINDED Conflict of Interest form (no author names used) should be submitted per manuscript with all author disclosures.

Charles P. Hannon, MD, MBA	[signature]	3/27/2025
Author Name (Print or Type)	Author Signature	Date
